# Vaginal Ovule Loaded with Bismuth Lipophilic Nanoparticles and Cetylpyridinium Chloride Inhibits Human Cervical Carcinoma and *Candida albicans* Growth

**DOI:** 10.3390/jfb15080206

**Published:** 2024-07-25

**Authors:** Claudio Cabral-Romero, Rene Hernández-Delgadillo, Jesús Alejandro Torres-Betancourt, Claudia María García-Cuellar, Yesennia Sánchez-Pérez, Juan Manuel Solis-Soto, Irene Meester, Nayely Pineda-Aguilar, Sergio Eduardo Nakagoshi-Cepeda, Juan Valerio Cauich-Rodríguez, María Argelia Akemi Nakagoshi-Cepeda

**Affiliations:** 1 Laboratorio de Biología Molecular, Facultad de Odontología, Universidad Autónoma de Nuevo León (UANL), Monterrey 66455, Nuevo León, Mexico; rene.hernandezdl@uanl.edu.mx (R.H.-D.); jesus.torresbtn@uanl.edu.mx (J.A.T.-B.); uanlsolis@gmail.com (J.M.S.-S.); sergio.nakagoshicp@uanl.edu.mx (S.E.N.-C.); maria.nakagoshicp@uanl.edu.mx (M.A.A.N.-C.); 2 Subdirección de Investigación Básica, Instituto Nacional de Cancerología, Ciudad de México 14080, Distrito Federal, Mexico; garcue57@gmail.com (C.M.G.-C.); s_yesennia@yahoo.com.mx (Y.S.-P.); 3 Departamento de Ciencias Básicas, Universidad de Monterrey, San Pedro Garza García 66238, Nuevo León, Mexico; elisabethd.meester@udem.edu; 4 Centro de Investigación en Materiales Avanzados, S.C. (CIMAV), Subsede Monterrey, PIIT, Apodaca 66628, Nuevo León, Mexico; nayely.pineda@cimav.edu.mx; 5 Centro de Investigación Científica de Yucatán, Unidad de Materiales, Mérida 97205, Yucatán, Mexico; juanvaleriocauich@hotmail.com

**Keywords:** antitumor and antimycotic activity, bismuth lipophilic nanoparticles (BisBAL NPs), cetylpyridinium chloride, human cervical carcinoma, topical treatment, vaginal ovule

## Abstract

Bismuth lipophilic nanoparticles (BisBAL NPs) and cetylpyridinium chloride (CPC) are antineoplastic and antimicrobial in vitro. As a next pre-clinical step, a clinically viable dosage form for vaginal application was developed. Compendial pharmacopeial tests (mass uniformity, disintegration, and compressive mechanics) and inductively coupled plasma optical emission spectroscopy were conducted on in-house developed glycerinated gelatin (60:15 *v*/*w*) vaginal ovules containing BisBAL NP-CPC. The antimycotic activity of BisBAL NP-CPC vaginal ovules was analyzed using disk diffusion and cell viability XTT assays. The antitumor properties of BisBAL NP-CPC vaginal ovules were assessed by cell viability MTT tests. BisBAL NP-CPC and drug-free vaginal ovules deposited into ex vivo porcine vaginas disaggregated without signs of adverse cytotoxicity within the timespan of clinical efficacy. BisBAL NP-CPC vaginal ovules demonstrated antifungal efficacy comparable to miconazole: *C. albicans* growth inhibition haloes in diffusion tests were 23 ± 0.968 mm (n = 3) for BisBAL NP-CPC and 20.35 ± 0.899 mm (n = 3) for miconazole. Likewise, BisBAL NP-CPC vaginal ovules reduced HeLa cell growth by 81%, outperforming the clinical reference of 500 μM 5-fluouracil, which induced a 70% growth inhibition. BisBAL NP-CPC incorporated into glycerinated gelatin vaginal ovules constitute an innovative drug delivery system for topical antimycotic and anti-cervical carcinoma treatments.

## 1. Introduction

Cervical cancer is the fourth most common cancer in women worldwide [1] and the most frequent type among women in developing countries [2]. Cervical cancer is categorized into three types: (i) squamous cell carcinoma (70–80% of new diagnoses), (ii) adenocarcinoma, and (iii) other epithelial cancers [3]. Cervical cancer is strongly associated with high-risk human papillomavirus (HPV) infection, which is considered the main etiological cause [4]. Risk factors include smoking, low socioeconomic status, and immunosuppression [5]. In Mexico, 9429 women were diagnosed with cervical cancer in 2020, with 9.2% aged between 20 and 40 years [6] and about two-thirds being HPV positive. Early sexual activity, multiple sexual partners, and several pregnancies are additional risk factors [7,8]. Over three-quarters of new cervical cancer cases are in advanced stages, with 16% in the early stage and 7% metastatic [9]. Mexico’s southern states have the highest mortality rates [8].

Conventional cytology (Papanicolaou) is the main diagnostic screening method. Treatment options include surgery, radiotherapy, ^192^Ir implants, and chemotherapy [4]. Furthermore, antiviral drugs, such as cidofovir, and immunomodulatory agents, such as interferon, have been suggested for treating HPV infections [10,11].

Nanomedicine, a relatively new discipline, develops “smart drugs” that target cancers without the severe adverse side effects of systemic chemotherapy. Other nanomedicine proposals are peptide-based nanovaccines against HPV infection [12]. Given the location of cervical cancer, a vaginal topical antitumor agent could deliver a high local concentration with minimal systemic exposure [13]. Nanocellulose and other nanocomposites, like liposomes and dendrimers, have been proposed as delivery carriers for antitumor agents. Altogether, nanoparticles hold promise for treating cervical and other cancers because of enhanced drug delivery, targeted therapy, and reduced side effects. Although significant advancements have been made, challenges related to scalability, reproducibility, long-term effects, and regulatory approval must be addressed.

We have reported consistent antitumor activity of bismuth lipophilic nanoparticles (BisBAL NPs) in in vitro assays and mouse models [14]. Bismuth is a biocompatible heavy semi-metal that is much cheaper than noble heavy metals such as gold and silver [15]. Bismuth(III) complexes are used clinically for diarrhea and heartburn, as well as for gastritis due to *Helicobacter pylori* in combination with an antibiotic However, bismuth offers an array of additional applications. It has been proposed as a contrast agent for X-ray imaging. Non-complexed bismuth nanoparticles outperformed both iodine- and barium-based contrast agents in mouse studies Because of its high atomic number, bismuth may serve as a radiosensitizer during anti-cancer radiotherapy. Additionally, several reports highlight the antimicrobial, antibiofilm, and fungicidal activities of BisBAL NPs against a variety of microbes in in vitro settings. Possible applications include water treatment, medical devices, and dentistry BisBAL NPs have been proven to possess anti-biofilm, antibiotic, antiparasitic, fungicidal, and anti-cancer properties with relatively low minimal inhibitory concentrations [16,17]. They can be synthesized relatively easily in various forms and shapes and coated onto surfaces with biological functions. Moreover, they are highly biocompatible and have low toxicity to healthy cells [15]. We have reported on a biodegradable hydrogel loaded with BisBAL NPs that inhibited the growth of several human cancers in pre-clinical studies [18].

Cetylpyridinium chloride (CPC) is an inexpensive cationic surfactant with a rapid broad-spectrum bactericidal and fungicidal effect against minor infections [19]. CPC is amphipathic; the positively charged hydrophilic region interacts with microbial surfaces, while the hydrophobic group integrates into the cell membrane, causing its disruption, the inhibition of cell growth, and ultimately cell death [20]. The membranes of cancer cells differ from their healthy counterparts in aberrant glycosylation, the overexpression of negatively charged molecules, and increased lipid rafts [21]. These changes make the neoplastic cell membrane more susceptible to quaternary ammonium surfactants, like CPC, than healthy cells. Indeed, we have reported the in vitro antitumor effect of CPC against human breast cancer cells and the cumulative antitumor effect of CPC and BisBAL NPs on human lung cancer cells [22,23]. Quaternary ammonium compounds like CPC are generally poorly absorbed by the oral route. CPC is widely used for local application in mouth washes, toothpastes, throat and nasal sprays, and lozenges, where absorption issues are irrelevant.

Thus, we have two inexpensive, biocompatible, wide-spectrum active ingredients that are already used clinically for other indications in oral and topical applications. Regarding cancer, BisBAL NP-CPC combination therapy had a cumulative antitumor effect on human lung cancer cells in vitro. Unlike vaginal infections, which are effectively treated with topical intravaginal creams or ovules, topical applications for cervical cancer treatment do not yet exist. To develop an innovative topical intravaginal treatment for cervical cancer, we developed a vaginal ovule to deliver the BisBAL NP-CPC combination treatment. We report on the essential pharmacopeial characteristics of the BisBAL NP-CPC-containing vaginal ovules and their efficacy in inhibiting *Candida albicans* and human cervical cancer (HeLa) cell growth in vitro.

## 2. Materials and Methods

### 2.1. Synthesis and Characterization of BisBAL NPs

BisBAL NPs were synthesized by the colloidal method according to Badireddy, in which bismuth nitrate was reduced by sodium borohydride and coated with mercaptoethanol [17]. A scanning electron microscope (FE-SEM; Nova NanoSEM 200, FEI Company, Hillsboro, OR, USA; 15 kV accelerating voltage) was employed to determine BisBAL NPs’ morphology, size, and distribution. The elemental chemical composition of BisBAL NPs was confirmed by SEM and energy-dispersive X-ray spectroscopy (EDX) (INCA X-Sight Detector, Oxford Instruments, Tubney Woods, UK), and a bismuth X-ray pattern was obtained with an X-ray diffractometer (Paralytical X´Pert PRO MRD, Great Malvern, UK) supplied with Cu Kα as an X-ray source (λ = 1.541874 Å).

### 2.2. BisBAL NP-CPC Suspension

A 1 mM CPC (Sigma-Aldrich; St. Louis, MO, USA) solution was mixed with a 50 mM BisBAL NP suspension in a 1:10 molar ratio using sterile bidistilled water to achieve the desired stock concentrations.

### 2.3. BisBAL NP-CPC Vaginal Ovule Development

Glycerinated gelatin (60:15, *v*/*w*) vaginal ovules were developed according to the Münzel method, a standard procedure for making vaginal ovules in the pharmaceutical industry [24]. To make a 100 mL BisBAL NP-CPC vaginal ovule solution, 15 g of hydrolyzed gelatin (Sigma-Aldrich; St. Louis, MO, USA) was dissolved in 30 mL of pre-heated (60 °C) sterile distilled water. Next, 60 mL of glycerol (Sigma-Aldrich; St. Louis, MO, USA) was added, followed by a 10 mL solution containing the active ingredients (0.1 mM CPC and 1 mM BisBAL NPs). The solution was mixed vigorously, poured into an ovule mold, and solidified at 4 °C for 30 min. Control vaginal ovules were drug-free.

### 2.4. Pharmacopeial Tests

#### 2.4.1. Mass Uniformity

The uniformity of mass was analyzed according to the European pharmacopoeia 2.9.5. Batches of vaginal ovules (n = 10) with or without active substances were weighed with an analytical balance (Ohaus, Ciudad de Mexico, D.F., Mexico). Inter- and intra-batch uniformity was determined.

#### 2.4.2. Disintegration Test and Bi Release Assay

The ability to deliver the active ingredients was analyzed using a disintegration test according to Carvalho [25]. Briefly, BisBAL NP-CPC vaginal ovules (n = 6) were immersed in 25 mL of one of the following solutions: sterile distilled H_2_O, 10 mM acetate buffer at pH 4.2, or 10 mM phosphate-buffered saline (PBS) at pH 7.4. The time needed for total fragmentation was recorded as the disintegration time. For free bismuth quantification, after standardized disintegration in PBS (37 °C or room temperature; 10, 20, or 60 min; n = 3), 1 mL samples were serially diluted (1:10) in sterile bidistilled water, and the amount of Bi was determined by inductively coupled plasma optical emission spectroscopy (ICP-OES) (ICP Spectrometer iCAP 6500 DUO; Thermo Electron Corporation; Rosemount, MN, USA: EE. UU).

#### 2.4.3. Mechanical Compression Test

One-week-old vaginal ovules, both those loaded with BisBAL NP-CPC and the drug-free controls (n = 12), were subjected to compression tests using a MiniShimadzu testing machine AG-I (Kyoto, Japan) applying a load of 1 kN at 1 mm/minute. The slope of the linear part of the load–displacement curve (100–150 N) was determined as an indication of compression characteristics.

### 2.5. Ex Vivo Mucoadhesion Assay

To estimate the dissolution capacity of BisBAL NP-CPC vaginal ovules within the female vagina, an ex vivo mucoadhesion assay was performed according to Teworte [24]. Post-slaughter porcine vaginas (n = 3) were cut into 4 cm pieces. A vaginal ovule (BisBAL NP–CPC or drug-free) was placed within a porcine vagina piece, which was fixed with a sterile clip and incubated in 25 mL of 30 mM acetate buffer (pH 4.2) for 3 or 9 h at 37 °C under aerobic conditions. After the incubation, ovule dissolution was verified and the porcine vagina mucosa morphology was analyzed for changes in coloration, bleeding, or signs of irritation.

### 2.6. Biological Tests

#### 2.6.1. Microbial Culture

*Candida albicans* (strain NCCLS67, ATCC 90029) was cultured in TSB agar or solution (TSB, BD DIFCO, Sparks, MD, USA) at 37 °C in aerobic conditions.

#### 2.6.2. Disk Diffusion Assay

The antimycotic activity of BisBAL NP-CPC vaginal ovules was determined with the disk diffusion assay [26]. Hereto, 100 μL of a *C. albicans* culture (0.5 MacFarland scale) was spread on TSB agar plates using a sterile cotton swab. Five-millimeter wells were punched into the agar plate and filled with the following treatment solutions: (i) BisBAL NP-CPC (1–0.1 mM; BisBAL NP-CPC vaginal ovules had been disintegrated previously as described above), (ii) drug-free PBS as a negative control, and (iii) 1% miconazole (Janssen-Zilag, Mexico City, Mexico) as a positive control of growth inhibition. Agar plates were incubated (18 h, 37 °C), and the halo inhibition zones were measured with a Vernier caliper. The experiment was conducted in triplicate.

#### 2.6.3. XTT Assay

The antimycotic activity of BisBAL NP-CPC vaginal ovules was further analyzed with the XTT (2,3-Bis-(2-methoxy-4-nitro-5-sulfophenyl)-2H-tetrazolium-5-carboxanilide) assay (Biotuim, Hayward, CA, USA). Hereto, 1 × 10^5^ *C. albicans* cells/100 μL TSB media were seeded into a 96-well plate and incubated for 24 h at 37 °C with the following treatments: 31, 62, 125, and 250 μM BisBAL NPs and 3, 6, 12, and 25 μM CPC (BisBAL NP-CPC vaginal ovules had been disintegrated previously as described above), drug-free PBS as a growth control, and 1200 μM miconazole as fungicide control. To evaluate cell survival, wells were incubated with 10 µL XTT/well for 2 h in the dark. Absorbance at 570 nm was measured with a spectrophotometer plate reader (BioTek, Winooski, VT, USA). The experiment was performed in triplicate.

#### 2.6.4. Cell Culture

The human cervicouterine adenocarcinoma cell line HeLa (ATCC, CCL-2, Rockville, MD, USA) was grown in Dulbecco’s modified Eagle’s medium/Ham’s F12 (DMEM/F12) supplemented with 10% fetal bovine serum (FBS; Gibco-Invitrogen, Carlsbad, CA, USA), penicillin (100 U/mL), streptomycin (100 μg/mL), and amphotericin B (0.25 μg/mL) (all antimicrobials from Sigma Aldrich) in cell culture flasks (Corning Inc., Corning, NY, USA) in a humidified atmosphere with 5% CO_2_. Confluent monolayers were detached with trypsin (Gibco-Invitrogen, Carlsbad, CA, USA), washed three times with PBS, and counted with a hemocytometer.

#### 2.6.5. Cell Viability MTT Assay

The possible inhibitory effect of BisBAL NP-CPC vaginal ovules on the HeLa cell line was analyzed with the in vitro MTT cell viability assay (Biotuim, Hayward, CA, USA) [27]. Hereto, HeLa cells (1 × 10^5^/100 μL media) were seeded into a 96-well plate, incubated for 24 h at 37 °C, and then treated with 31, 62, 125, and 250 μM BisBAL NP-CPC (BisBAL NP-CPC vaginal ovules had been disintegrated previously as described above), 500 μM 5-fluorouracil (5-FU, positive control of growth inhibition) (Sigma-Aldrich; St. Louis, MO, USA), or drug-free PBS as a negative control for 24 h at 37 °C and 5% CO_2_. Exposure to the drugs was stopped by washing the monolayer with cold PBS. According to the provider´s instructions, 100 μL dimethyl sulfoxide (DMSO) was added to each well to dissolve the formazan crystals. Absorbance was measured at 570 nm with a spectrophotometer microplate absorbance reader (Biotek, Winooski, VT, USA). The assay was performed in triplicate.

### 2.7. Statistical Analysis

The normality of the data was verified with the Shapiro–Wilk test. A significant difference between two or more parametric groups was evaluated with a Student´s *t*-test or a one-way analysis of variance (ANOVA) and Tukey HSD test, respectively. The level of significance (α) was set at 0.05.

## 3. Results

### 3.1. BisBAL NP and BisBAL NP-CPC Vaginal Ovule Characterization

**Figure 1:**
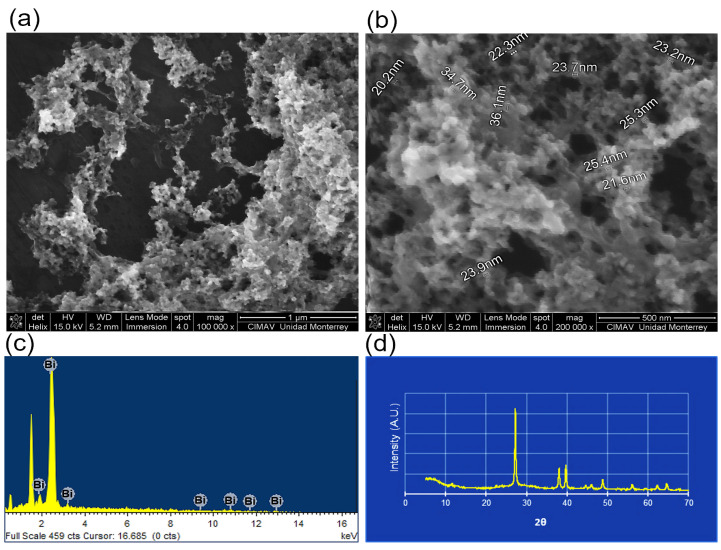
Field emission scanning electron microscopy (FE-SEM) of bismuth lipophilic nanoparticles (BisBAL NPs). BisBAL NP micrography (**a**,**b**). Chemical composition and bismuth identity were corroborated by energy-dispersive X-ray spectroscopy (**c**) and the X-ray diffractometry pattern (**d**).

**Figure 2:**
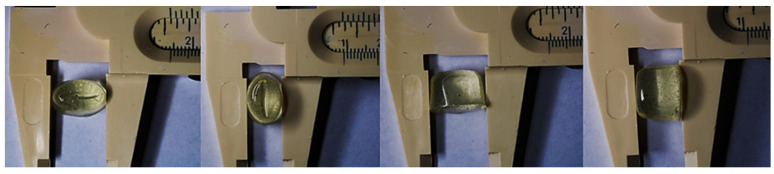
Morphology of BisBAL NP-CPC vaginal ovules. The shape and size of vaginal ovules containing BisBAL NP-CPC was determined with a Vernier.

**Figure 3:**
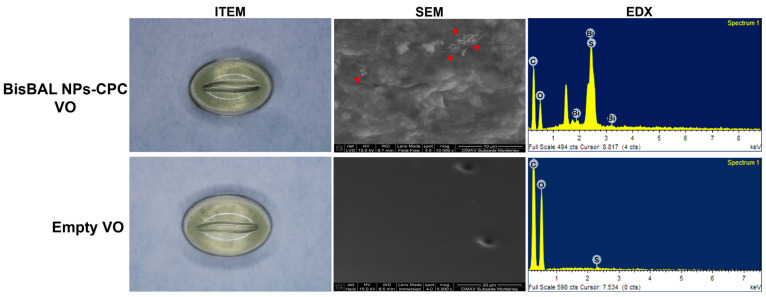
Characterization of vaginal ovules containing BisBAL NPs and CPC. Drug-free and BisBAL NP-CPC-loaded vaginal ovules (BisBAL NPs-CPC VO) were observed by field emission electron microscopy (FE-SEM), and the chemical elemental composition was obtained by energy-dispersive X-ray spectroscopy (EDX). Red arrows show the presence of BisBAL NPs on VO.

BisBAL NP spheres had an average diameter of 25.6 ± 9.31 nm (Figure 1) and formed electron-dense aggregates in which bismuth’s presence was corroborated by EDX-spectra (Figure 1). The oval-shaped ovules (1.5 × 1 × 1 cm) were translucent (Figure 2), except for black dots due to the BisBAL NPs. Bismuth presence was confirmed by SEM and EDX peaks only in the BisBAL NP-CPC vaginal ovule samples (Figure 3).

### 3.2. Mass Uniformity

**Figure 4:**
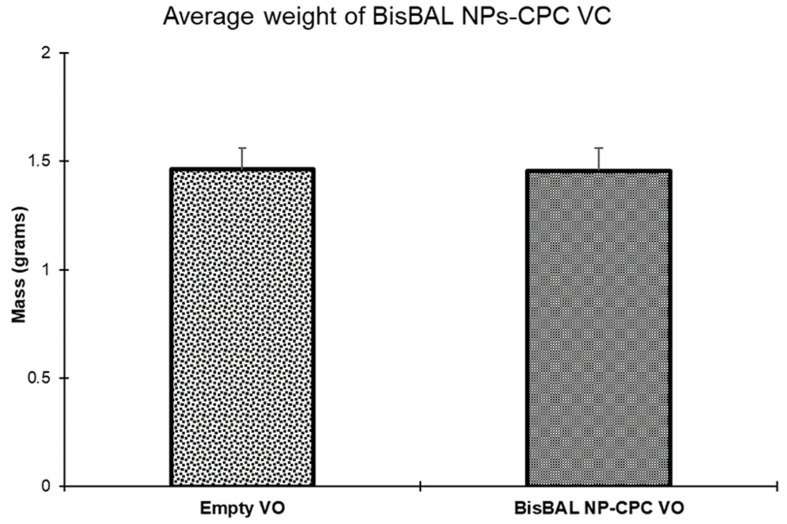
BisBAL NP and CPC-containing vaginal ovules had the same mass uniformity as the drug-free vaginal ovules (VO) (n = 10). Bars and error bars, mean ± SD.

Both BisBAL NP-CPC and drug-free vaginal ovules weighed 1.5 ± 0.105 g, complying with the European Pharmacopoeia standard (less than 10% of the individual mass deviates from the average mass by 5%). Similar mean and SD values suggest that the inclusion of BisBAL NP-CPC into a vaginal ovule did not affect the final weight (Figure 4). Thus, the vaginal ovules complied with mass uniformity.

### 3.3. Disintegration Test

The disintegration time of vaginal ovules containing BisBAL NP-CPC was 19 min in the acetate buffer of pH 4.2 that simulated the physiological environment of the vagina. The physiological control environment, PBS at pH 7.4, had the longest disintegration time (23 min), while water was the most efficient (14 min) (Table 1). Drug-free vaginal ovules disintegrated in similar time spans, suggesting that BisBAL NP-CPC incorporation into the vehicle did not affect the disintegration properties of the delivery vehicle.

### 3.4. Drug Release Test

**Figure 5:**
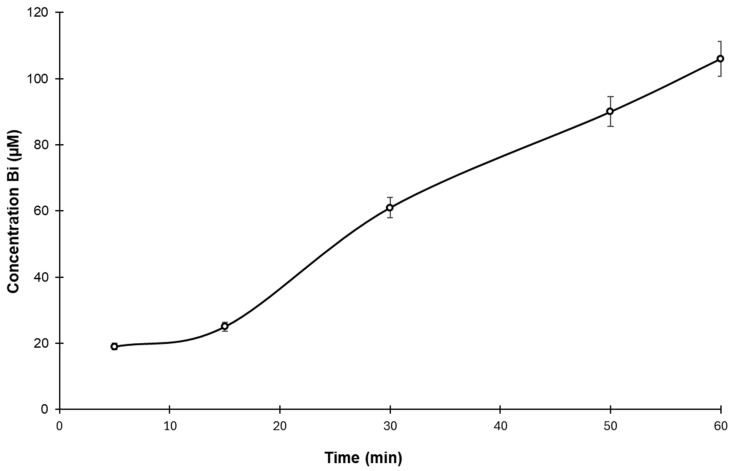
Bismuth release from disintegrating BisBAL NP-CPC vaginal ovules. Free bismuth (Bi) was determined by ICP-OES (n = 5).

The amount of free bismuth released from disintegrated BisBAL NP-CPC vaginal ovules increased in a time-dependent manner, reaching its maximum value (100 µM) at the last time point (60 min) (Figure 5). This result corroborates the effectiveness of bismuth delivery once the vaginal ovules have been disintegrated.

### 3.5. Mechanical Compression Test

**Figure 6:**
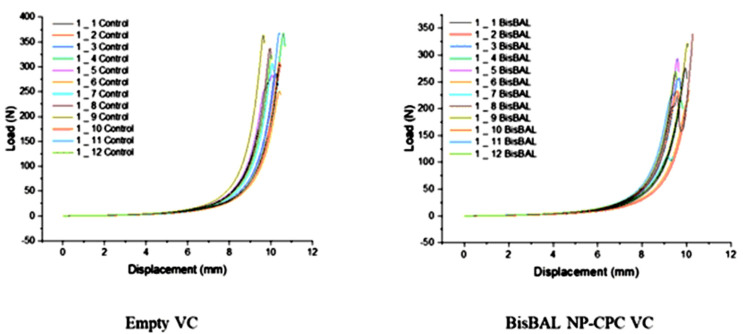
Load–−displacement profiles of BisBAL NP-CPC and drug-free vaginal ovules. Mechanical compression assays were run at 1 kN at 1 mm/min (n = 12) and the slope was determined from the linear part of the corrected zero displacement point.

The load–displacement curves of BisBAL NP-CPC-containing and drug-free vaginal ovules were similar, with mean slope values of 139.64 ± 8.84 N/mm and 143.32 ± 3.351 N/mm, respectively (n = 12; *p* = 0.1634) (Figure 6). Therefore, it seems that BisBAL NP and CPC incorporation into vaginal ovules did not alter their mechanical properties.

### 3.6. Ex Vivo Mucoadhesion Test

**Figure 7:**
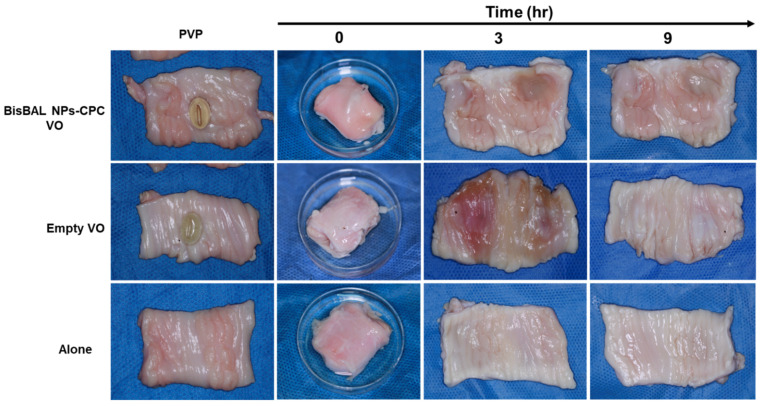
Ex vivo mucoadhesion test of BisBAL NP-CPC vaginal ovules (VO). Pieces of porcine vagina were used to analyze the disintegration of BisBAL NP-CPC VO (n = 3) in a vaginal ambient (37 °C; pH 4.2; acetate buffer). Controls included drug-free vaginal ovules and ovule-free porcine vagina pieces. After 3 and 9 h, pieces of porcine vagina were opened and observed.

BisBAL NP-CPC and drug-free vaginal ovules disaggregated completely within 3 h in the porcine vagina model. There were no signs of irritation, damage, or bleeding of the vaginal mucosa tissue at either 3 h or 9 h (Figure 7). In all cases, the porcine vagina pieces, whether in contact with any type of vaginal ovule or not, showed healthy tissue, suggesting the absence of toxicity.

### 3.7. Antimycotic Activity

**Figure 8:**
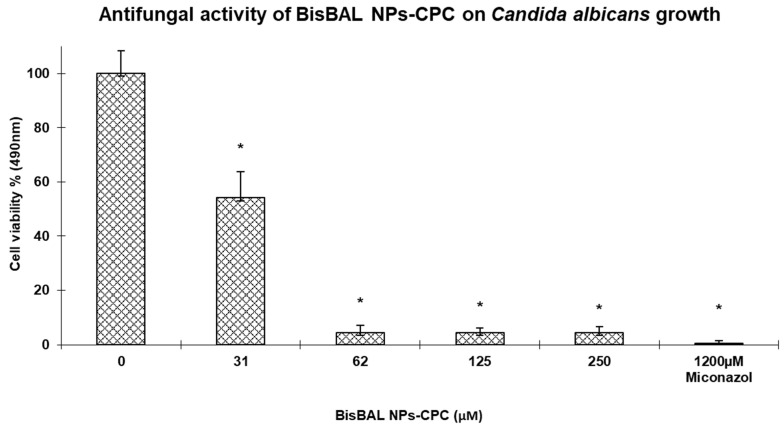
Antifungal activity of BisBAL NP-CPC vaginal ovules on *C. albicans* growth by XTT assay. Antifungal activity was significantly reduced with the BisBAL NP-CPC treatment compared to controls. Bars, mean ± SD (n = 5); *, statistically significant (*p* < 0.0001).

The potential of BisBAL NP-CPC vaginal ovules to inhibit the fungal growth of *C. albicans* was evaluated by the disk diffusion assay. BisBAL NP-CP vaginal ovules caused an inhibition halo of 23 ± 0.968 mm (n = 3), whereas the halo was 20.35 ± 0.899 mm (n = 3) for 1200 μM miconazole, the positive control of inhibition (Table 2). The antimycotic activity of BisBAL NP-CPC was confirmed with an XTT cell viability assay. *C. albicans* growth was reduced by 47% and 93% after a 24 h exposure to BisBAL NP-CPC at 31 ± 3.1 μM and 62 ± 6.25 μM, respectively (Figure 8). The positive control of growth inhibition (1200 μM miconazole) abolished fungal growth. Thus, our results confirmed the antimycotic potential of BisBAL NP-CPC liberated from disintegrated ovules.

### 3.8. Antitumor Activity

**Figure 9:**
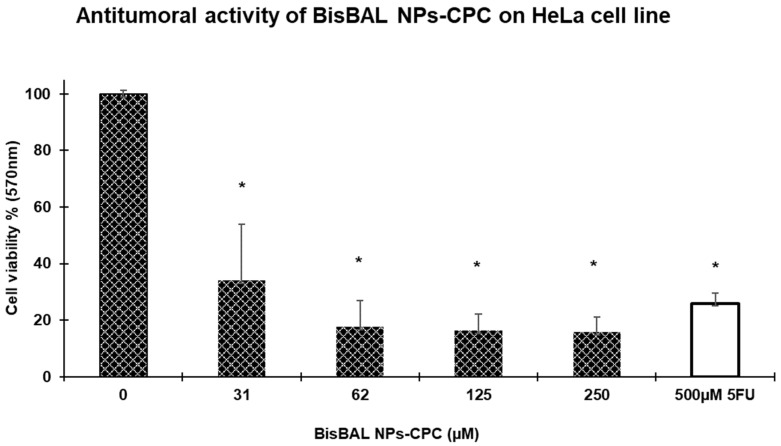
Antitumor activity of BisBAL NP-CPC vaginal ovules on HeLa cell growth by cell viability MTT assay. Antitumor activity was significantly reduced with BisBAL NP-CPC treatments (250 ± 31 µM) compared to the control group (0 µM). Error bars show mean ± SD (n = 5), *, statistically significant (*p* < 0.0001).

The antitumor activity of BisBAL NP-CPC vaginal ovules was explored by MTT cell viability on HeLa cells, as previously described [18]. HeLa cell growth diminished 63% after exposure to a BisBAL NP-CPC (31–3 μM) solution that had been obtained from a disintegrated vaginal ovule, and 81% after exposure to a similar solution containing 62 μM of BisBAL NPs and 6.25 μM of CPC, while the positive control of tumor cell inhibition, 500 μM of 5-FU, reduced tumor cell growth by 70% (Figure 9).

## 4. Discussion

Despite the availability of a safe and effective HPV vaccine as a preventive measure for cervical cancer for nearly two decades [28,29,30], cervical cancer remains a significant challenge in modern medicine worldwide. Treatment for cervical cancer typically involves surgery, radiotherapy, and/or chemotherapy. Recently, our group reported that BisBAL NPs selectively inhibit HeLa cell growth at a concentration of 1 μM [18].

While vulvovaginal candidiasis is effectively treated with topical drugs like ovules or creams [31,32], no such topical treatments exist for cervical cancer. Vaginal drug delivery offers several advantages over oral administration, including (i) a higher dose of the active ingredient at the targeted microenvironment, (ii) a larger therapeutic effect at the tumor site, (iii) avoidance of metabolic transformation by the liver, and (iv) reduced or no exposure of non-tumor tissues to the drugs [33]. Various vaginal drug delivery systems exist, such as gels, ovules, and tablets composed of mucoadhesive polymers, which are user-friendly and well accepted by women [34,35]. The localized delivery of antitumor drugs through vaginal administration can result in fewer systemic side effects, thereby improving patient compliance and outcomes.

Nanomedicine, a relatively new discipline, aims to develop drug carriers that improve the controlled delivery of target-specific antitumor agents, increasing effectiveness and reducing adverse side effects [36,37,38,39]. Innovative nanomaterials have the potential to enhance the efficacy of cervical cancer chemotherapy [2]. Here, we propose a topical anti-cervical cancer treatment using glycerinated gelatin vaginal ovules containing BisBAL NPs and CPC as active ingredients. Our innovative formulation consists of translucent oval-shaped ovules with black dots (BisBAL NP) and dissolved CPC. The ovules are of appropriate size and shape for vaginal delivery [40]. Vaginal ovule batches had a uniform mass of 1.5 ± 0.105 g. The vaginal ovules disintegrated in an acetate buffer (pH 4.2) in 19 min, in PBS (pH 7.4) in 23 min, and in water in 14 min. Other studies have reported similar disintegration rates for different vaginal ovule compositions [24], indicating the suitability of our formulation for clinical application.

The glycerinated gelatin vaginal ovules and lipophile-coated nanostructures demonstrated excellent potential for bismuth delivery, achieving the highest bismuth levels within an hour. In contrast, another study reported that platinum releasing from its nanoparticle vehicle took 10 h [41]. Furthermore, we confirmed the disintegration of BisBAL NP-CPC vaginal ovules through an ex vivo mucoadhesion assay on porcine vaginas, simulating the natural vaginal environment. The active ingredients were released within 3 h in the humid environment (pH 4.2, 37 °C). Interestingly, 9 h of exposure to a BisBAL NP-CPC vaginal ovule did not cause obvious irritation, damage, or bleeding in vaginal tissue. In contrast, treatment with Cerviron^®^ vaginal ovules caused minor bleeding in 33.91% of patients [42].

Regarding drug efficacy, BisBAL NP-CPC vaginal ovules caused an inhibition halo of 23.0 ± 0.97 mm in *C. albicans* cultures, surpassing the 20.35 ± 0.90 mm inhibition halo of miconazole. XTT assays confirmed the antimycotic activity, showing a 94% reduction in fungal growth when exposed to a solution containing 62 µM of BisBAL NPs and 6.2 µM of CPC liberated from disintegrated vaginal ovules. Various alternative formulations for treating candidiasis have been reported, such as chitosan-hydroxypropyl methylcellulose films [43], itraconazole-loaded vaginal bioadhesive films [44], and fenticonazole-containing intravaginal ovules [45].

The antitumor properties of BisBAL NP-CPC vaginal ovules were evaluated on the human cervical carcinoma HeLa cell line. An in vitro 24 h exposure to 31 µM of BisBAL NPs/3.1 µM of CPC reduced HeLa cell growth by 62%, while exposure to 62 µM of BisBal NPs/6.2 µM of CPC reduced growth by 81%. In comparison, 500 µM of 5-FU reduced HeLa cell growth by 70%. There are no previous reports on using vaginal ovules as antitumor vehicles in cervical cancer patients. However, our previous studies have shown that a 1 h exposure to a BisBAL NP-loaded biodegradable hydrogel inhibited cervical cancer cell growth [18]. Given that the squamous cell carcinoma type is not only the most common cervical cancer but also the most accessible for topical treatment, drug-loaded vaginal ovules could be an innovative delivery system for locally inhibiting cervical cancer. This gap in the current treatment landscape highlights a significant opportunity for developing novel therapeutic strategies. To our knowledge, despite the clear advantages, there are no topical vaginal delivery proposals for cervical cancer within the realm of nanomedicine [46,47,48].

Considering the high acceptance of vaginal ovules by women [28,29], their use for topical, targeted drug delivery with less systemic adverse side effects could improve treatment compliance and outcome. The low cost of our formulation broadens treatment accessibility in lower-income countries.

## 5. Conclusions

Glycerinated gelatin vaginal ovules containing BisBAL NPs and CPC as active ingredients provide an innovative, low-cost, and effective topical alternative treatment for cervical cancer. This formulation not only offers a targeted antitumor therapy but also an antimycotic treatment, offering dual efficacy. The local delivery of the active ingredients ensures a high concentration at the tumor or infection site, enhancing therapeutic outcomes while minimizing systemic exposure and associated adverse side effects. Moreover, the ease of use of vaginal ovules makes them a practical option for patients, promoting better compliance with the treatment regimen. Additionally, the cost-effectiveness of this formulation makes it accessible to a broader population in resource-limited settings. Thus, this smart innovation promises to create a significant advancement in cervical cancer therapy.

## Figures and Tables

**Table 1:** Disintegration of vaginal ovule (VO) containing BisBAL NPs and CPC (n = 6).

Dissolution Medium	Disintegration Time (min) *
Acetate buffer (pH, 4.2)	19 ± 0.482
PBS (pH, 7.4)	23 ± 0.788
Distilled water	14 ± 0.842

* Mean ± SD.

**Table 2:** Antimycotic values for BisBAL NP-CPC VO against *Candida albicans*.

Empty VO	1 mM BisBAL NP-CPC VO	24 mM Miconazole
0 ± 0	23 ± 0.968	20.35 ± 0.899

Values are mean ± SD (n = 3); significance level, α = 0.05.

## Data Availability

The original contributions presented in the study are included in the article, further inquiries can be directed to the corresponding author.
